# Human challenge trials to assess the efficacy of currently approved COVID-19 vaccines against SARS-CoV-2 variants

**DOI:** 10.1080/22221751.2021.1896956

**Published:** 2021-03-13

**Authors:** Shan Su, Yiming Shao, Shibo Jiang

**Affiliations:** a Key Laboratory of Medical Molecular Virology (MOE/MOH/CAM), School of Basic Medical Sciences, Shanghai Institute of Infectious Diseases and Biosecurity, Fudan University, Shanghai, People’s Republic of China; b State Key Laboratory of Infectious Disease Prevention and Control (SKLID), National Center for AIDS/STD Control and Prevention (NCAIDS), Chinese Center for Disease Control and Prevention (China CDC), Collaborative Innovation Center for Diagnosis and Treatment of Infectious Diseases, Beijing, People’s Republic of China

Human challenge trials (HCTs) are trials in which volunteers are intentionally challenged with an infectious organism, such as virus, prior to, or post-, vaccination [1].

In 1796, physician Edward Jenner inoculated an eight-year-old boy with cowpox sample and deliberately exposed him to human smallpox, resulting in the creation of the first-ever vaccine in human history. This uncontrolled trial with a deadly pathogen would, of course, be ethically unacceptable today. Currently, HCTs are generally restricted to pathogens that have low fatality rate with efficacious treatments to reduce or eliminate mortality, such as influenza virus, dengue virus, respiratory syncytial virus, and common human coronaviruses.

To further speed up the development of COVID-19 vaccines, 35 members of the US House of Representatives called upon US regulators to allow HCTs for COVID-19 vaccines in April of 2020, and 177 prominent scientists, including 15 Nobel laureates, advocated for HCTs in July of 2020 [2]. The UK government signed a contract to develop a COVID-19 HCT on 20 October 2020 [3] and received ethics approval on 17 February 2021 [4]. However, many scientists still believe that it is unethical to use HCTs for COVID-19 vaccine development [5], especially in view of the fact that several safe and effective COVID-19 vaccines have already been approved for general, or emergency, use against wild-type SARS-CoV-2.

Here, however, we are proposing the use of HCTs to rapidly evaluate the efficacy of currently approved COVID-19 vaccines against SARS-CoV-2 variants, including B.1.1.7 (UK), B.1.351 (South Africa) and B.1.1.248 (Brazil). Although some reports have shown that the antibodies from persons who received the COVID-19 vaccines have similar, or moderately reduced, level of neutralizing activity against these variants *in vitro*, no valid evidence from clinical trials supports these conclusions. South Africa suspended its Oxford/AstraZeneca COVID vaccine roll-out on 7 February 2021, after a statement received from the University of Witwatersrand, site of the AZD1222 *vaccine* clinical trial in Johannesburg, indicated that the vaccine “provides minimal protection against mild-moderate COVID-19 infection” by the now dominant South African variant B.1.351 [6]. In a conventional phase-III clinical trial, subjects will quickly resume their usual life after vaccination. As such, the percentage of subjects exposed to the virus depends on such factors as viral transmissibility and pathogenicity, local morbidity rate, government constraint, and personal hygiene. Such trial requires a large number of subjects to yield a statistically significant efficacy profile. For example, the phase-III trial of mRNA-1273 (NCT04470427) and BNT162b2 (NCT04368728) enrolled 30,000 and 43,998 participants, respectively. In contrast, participants of HCTs can be exposed to maximal viral attack, thus requiring a much smaller number of subjects. In the COVID-19 HCT guidebook drafted by the WHO Advisory Group, it was estimated that 28 analysable volunteers would be required in each trial arm if a vaccine candidate of 65% efficacy is to be tested in a controlled human infection model (CHIM) with an approximate 70% attack risk [7].

The HCT model herein proposed is intended to minimize risks to subjects and maximize benefits. Therefore, HCT participants should be young (20- to 29-year-olds) and healthy because the estimated case fatality ratio (CFR) of SARS-CoV-2-infected 20- to 29-year-olds in China is 0.03%, about 21-fold lower than the CFR (0.657%) of the overall infected patients (0- to ≥80-year-olds) [8,9]. The CFR is expected to be further reduced if subjects enrolled in HCTs receive a vaccine with >50% efficacy before viral challenge.

HCTs accelerate vaccine development because they can eliminate the time it takes for natural infection to run its course. Also, the reduced number of enrolees in HCT, when compared to the classical phase-III clinical trials, could significantly shorten recruitment time, thus further reducing the time and budget for clinical trials.

Although it is reported that variants B.1.1.7 (UK), B.1.351 (South Africa) and B.1.1.248 (Brazil) have increased transmissibility, no clear evidence has emerged to suggest that these variants are associated with more severe disease outcome [10]. This means that no significant increase of the risk to HCT participants is expected.

In general, the optimal challenge dose of a virus to be used for HCTs of a vaccine has to be determined in unvaccinated individuals, in order to select an optimal challenge dose of a virus having a relatively high attack rate, while limiting disease severity. As proposed by the WHO’s Advisory Group, an initial group of few volunteers (1–3) will be challenged with ∼1 × 10^2^ TCID50 SARS-CoV-2. If the safety profile is acceptable, the second group of more subjects (3–5) will be inoculated with the same dose of the virus. If the safety profile in the first two groups is acceptable, an additional 5–10 subjects will be challenged with the same dose of the virus. Then, dose escalation to ∼1 × 10^3^ TCID50 will proceed with increasing numbers of subjects if the safety profile is acceptable [7,11]. To avoid unnecessary risk to these unvaccinated individuals, we recommend the use of non-human primates (NHPs) for the primary determination of the optimal challenge dose of the virus to be tested, as the results from NHP experiments are relatively more useful for predicting human outcomes [12]. The final decision could be made based on the results from the UK HCT, which will begin within a month. The participants in the control group should also receive the approved vaccine to be evaluated in the HCT before being challenged with SARS-CoV-2 wild-type strain, based on which the vaccine was designed.

Highly effective anti-coronavirus drugs or COVID-19 therapeutics are still not on the horizon, but the application of systemic corticosteroids, or combining the neutralizing antibodies bamlanivimab and etesevimab, is expected to reduce the risk of hospitalization and death from COVID-19. In a phase III clinical trial, CD24Fc was shown to reduce the risk of respiratory failure or death by more than 50% [13]. If these drugs are obtainable by physicians who conduct the HCTs, they should be on hand before the start of a COVID-19 HCT.

In summary, the emergence of SARS-CoV-2 variants with altered transmissibility, pathogenesis, virulence, or a combination of these, has cast a shadow over COVID-19 vaccine development. Antibodies in convalescents and vaccinees may further exert selective pressure on wild-type SARS-CoV-2, resulting in complete escape of the virus from the current vaccines [14]. Therefore, in order to keep pace with the continuous emergence of SARS-CoV-2 variants as they are sequenced and validated, HCTs have more benefits than regular clinical trials. First, HCTs can quickly test whether or not the approved vaccines can protect people against the new variants of SARS-CoV-2. Second, HCTs can accelerate testing and development of next-generation COVID-19 vaccines, including those protect against asymptomatic SARS-CoV-2 infection. Third, HCTs better demonstrate viral pathogenesis by both natural disease course and immune response because of the precise timing of infection. Fourth, HCTs can better discern the correlation between immune responses, including both IgA and IgG antibodies with or without neutralizing activity as well as T cell immune response, to vaccine candidate and protection or, importantly, enhancement of disease, because the immune responses are closely measured post-inoculation. Fifth, HCTs can predict the duration of vaccine-induced immunity, whereas the phase-III field trial would be terminated once infected cases reach a set point. The knowledge on the duration of immune responses will help decide the time for vaccine boost, which is needed to maintain the herd immunity in the population. Sixth, by using the same established CHIM, HCTs can be used to compare the efficacy of different vaccine candidates.

Therefore, the main proposal here is that the HCT can shorten the time for evaluating the protection rate of a previously approved vaccine against a SARS-CoV-2 variant. If the approved vaccines are still effective, the public’s concerns can be allayed. If they become ineffective against these variants, new vaccines can be quickly designed and evaluated with COVID-19 HCTs, finally resulting in the approval and production of an effective and safe “updated” vaccine long before the next, potentially more virulent, SARS-CoV-2 variant spreads around the world.

As pointed out by Drs. Plotkin and Caplan, “extraordinary diseases require extraordinary solutions” [9].
